# The effects of HIV self-testing kits in increasing uptake of male partner testing among pregnant women attending antenatal clinics in Kenya: a randomized controlled trial

**DOI:** 10.11604/pamj.2019.33.213.14160

**Published:** 2019-07-16

**Authors:** Tom Marwa, Sarah Karanja, Justus Osero, Alloys Orago

**Affiliations:** 1 School of Public Health, Kenyatta University, Nairobi, Kenya; 2 Monitoring, Evaluation and Research Unit, Amref Health Kenya, Nairobi, Kenya; 3 Faculty of Medicine, Department of Pathology, Kenyatta University, Nairobi, Kenya

**Keywords:** HIV self-testing, ANC mothers, male partners, drivers and barriers, randomized controlled trial, male involvement

## Abstract

**Introduction:**

HIV self-testing could add a new approach to scaling up HIV testing with potential of being high impact, low cost, confidential, and empowering for users.

**Methods:**

Pregnant women attending antenatal clinics (ANC) and their male partners were recruited in 14 clinics in the eastern and central regions of Kenya and randomly allocated to intervention or control arms at a ratio of 1:1:1. Arm 1 received the standard of care, which involved invitation of the male partner to the clinic through word of mouth, arm 2 received an improved invitation letter, and arm 3 received the same improved letter and, two self-testing kits. Analysis was done using adjusted odds ratios (aOR) at 95% confidence intervals (CI) to calculate and determine effects of HIV self-testing in increasing uptake of male partner testing.

**Results:**

A total of 1410 women and 1033 men were recruited; 86% (1217) women and 79% (1107) couples were followed up. In arm 3, over 80% (327) of male partners took HIV test, compared to only 37% (133) in arm 2 and 28% (106) in arm one. There was a statistical significance between arm one and two (p-value=0.01) while arm three was statistically significant compared to arm two (p-value<0.001). Men in arm three were twelve times more likely to test compared to arm one (aOR 12.45 (95% CI 7.35, 21.08)).

**Conclusion:**

Giving ANC mothers test kits and improved male invitation letter increased the likelihood of male partner testing by twelve times. These results demonstrate that HIV self-test kits could complement routine HIV testing methods in the general population.

## Introduction

Sub-Saharan Africa accounts for over 70% of all HIV related morbidity and mortality globally while in 2016, 27 million of the 36.7 million People Living with HIV (PLHIV) were in sub-Saharan Africa. Kenya has the fourth largest HIV epidemic globally. In 2012 alone, an estimated 1.6 million people were living with HIV and close to 58,000 people died of HIV related illness [1]. HIV and AIDS prevalence peaked at 11% in the mid-nineties and has since dropped and stabilized to below 5.6% in 2012 due to mainly rapid scale up of various HIV interventions that included HIV care and treatment, Prevention of Mother to Child Transmission (PMTCT), Voluntary Medical Male Circumcision (VMMC), HIV Testing and Counselling (HTC) among other evidence based interventions [2]. Kenya has a generalized epidemic, meaning it affects all sections of society including children, young people, adults, women and men. HTC is a very effective strategy in linking those infected with HIV into care and treatment and making appropriate referrals to other HIV support services [3]. Kenya has adopted a number of strategies including provider initiated testing and counselling (PITC), outreach testing and counselling, home based testing and counselling. Other strategies that have proved effective are integration of HTC in antenatal care and reproductive health [4]. Kenya has in the recent years adapted and now included in the national HIV Testing Services (HTS) guidelines, HIV self-testing approach which is defined as any form of testing in which an individual collects his or her own sample then performs a simple HIV test; and is, therefore, the first to know the results. Self-testing could add a new approach to support scaling up testing with a potential to be high impact, low cost, confidential, and empowering for users. Unregulated sales of test kits and evidence of informal self-testing by health workers and community health workers indicate a demand for self-testing. Out-of-facility approaches to offering testing in the community and the workplace are means of bringing access to testing closer to clients [3, 5]. Distance has always been cited as a barrier to HIV testing and, these methods could be complementary means to scale up HIV testing. There is ample evidence documenting the impact of male involvement on the various components of PMTCT and HTS programs [6]. Men play an important role in reducing women's risk of acquiring HIV [7], women's utilization of services, including testing for HIV [8] and in promoting a woman's health [9]. Male partners also influence women's treatment decisions, including whether she receives medication [10] and whether she adheres to infant feeding advice [11].

Many women in Kenya fear disclosing their HIV positive status to their male partners because of stigma or discrimination, many at times these same women will end up experiencing some form of physical or psychological violence, and/or even death [12]. Thus, the potential for violence as a consequence of disclosure remains an important question to address empirically. Uptake of facility-based HIV testing among pregnant women attending ANC is as high as 88% [1], but approaches to promote male involvement during ANC services, such as having male-only clinics on special days or evening clinics have been tried without much success [13]. There is limited information on male involvement in HTC at the antenatal setting in the developing world [14]. Correlates of male HIV testing behavior and the reasons for obtaining HIV tests have been examined in sub-Saharan Africa using population-based studies and within workplace and clinic-based HTC trials [15]. Reporting HIV risk behavior patterns generally appeared to be associated with increased testing behavior, but with varying socio-demographic characteristics across settings. Eastern and central regions of Kenya have the lowest testing rates among male partners in Kenya [2]. Stigma and discrimination is also widespread in these regions, which is a barrier to seeking HIV services among those who test positive. [1]. Women feel burdened by clinics' request for their partner to visit health facilities for testing [16]. Fear of disclosing HIV results to male partners results into many ANC women feeling less empowered to ask their partners to undergo an HIV test, rather, women prefer the request come from the clinic staff themselves [6]. The current nationwide practice in Kenya to encourage male involvement is by sending a letter or word of mouth home with the pregnant woman, regardless of her HIV status, which invites her partner to come to the facility for involvement in ANC. The letter does not mention the need for HIV testing of the partner, nor does it contain important and potentially motivating information such as the rate of married individuals who are HIV-positive but have an HIV-negative partner in Kenya i.e. discordance rate (45%). The KAIS 2012 report showed that 71% of Kenyans indicated a willingness to use an HIV self-test kit (74.1% of males and 67.3% of females) [1]. The aim of this study was to determine the effects of HIV self-testing kits on uptake of male partner testing among pregnant women attending antenatal clinics in Kenya.

## Methods

**Trial design:** this was a three-arm open, individually randomized controlled trial (1:1:1), parallel-group study conducted in 14 sites in Meru, Kiambu, Kitui, Embu and Murang'a counties of Kenya. The study was approved by the Kenyatta university ethics review board and Kenya National Commission for Science, Technology and Innovation (NACOSTI). The study was conducted between October 2015 and April 2017.

**Participants:** the primary study participants were women attending ANC clinic for the first time (first ANC visit in their current pregnancy). The 2012 Kenya AIDS Indicator Survey showed that 44% of HIV positive married/cohabiting persons were in discordant relationship [1].

**Inclusion criteria expectant women:** first ANC visit in this pregnancy; 18 years and above.

**Exclusion criteria expectant women:** had no current male partner/does not have at least weekly contact with partner; woman reported that her partner is HIV positive and on HIV care; woman reported that her partner has tested within last three months; woman concerned for her safety or feel at risk of Gender Based Violence (GBV) if she asked her partner to self-test, or woman currently does not feel safe at home to encourage HIV testing. The study excluded women only if the GBV risk was directly associated with discussion of HIV testing with their partner. This exclusion criterion was administered prior to randomizing women into the study arms.

**Inclusion criteria male partner:** male partner of ANC client enrolled in the study; had a personal contact with the woman at least once per week; has good cognitive abilities and mentally sound to respond to the survey questions.

**Exclusion criteria male partner:** unwilling to participate in the study.

**Study site:** fourteen health facilities were purposively selected from 1 mission and 180 public health facilities in Meru, Embu, Kiambu, Kitui and Murang'a counties. The selection was based on the following reasons: good social support mechanism for the pregnant mothers e.g. the availability of mentor mothers groups, HIV psychosocial support groups, to mitigate other unwanted outcomes such as GBV; and other crucial services for male partners who test positive and need immediate support among other services; high volume facilities with support from HIV/AIDS implementing partners. These organizations were critical in providing HIV support, care treatment and referrals.

**Intervention:** arm 1 (control 1) received the current standard of HIV testing and counseling services offered to Kenyan ANC clients, which include male partner invitation to come to the ANC clinic with his pregnant partner and this was done through word of mouth a brief invitation letter inviting the male client to the clinic. The invitation letter does not mention HIV testing but highlights the need by the male partner to come and discuss the health of the wife/partner. Arm 2 (control 2) received an improved letter that described the benefits of HIV prevention and testing and was meant to invite the partner to the clinic for standard HIV testing. The rationale for inclusion of this control group was to isolate the impact of self-testing kit availability, and control for the additional information that must be provided to test-kit recipients. Arm 3 (intervention arm) were given the same improved letter as arm 2 describing the benefits of HIV testing. Participants were also given demonstrations on how to use the oral test kits then they were provided with two oral self-test kits, counseling, communications materials and clear instructions on how to: self-test, introduce the self-test kit to their partners and report use of tests through mobile phones or during their next ANC visits. Participants in all three-study arms received more counseling on PMTCT, linkages to care and support groups if tested positive, and counseling on partner testing in general. All clients were informed how they would be followed up with either SMS messages or one on one by the study assistants where mobile use was not feasible. They were also given information on who to call if, for example, they had questions on the HIV self-testing, what the results meant or resources for GBV.

**Outcomes:** the primary outcome was the proportion of male partners who tested for HIV within three months as reported by the female partners. The secondary outcomes were acceptability of self-testing by the male partner, incidence of GBV, linkage to care and man's self-report of HIV testing to supplement the woman's self-report, and measure concordance in reporting within the couple.

**Sample size:** sample size was calculated by comparing study arm one and study arm two, and study arm two and study arm three. Sample size calculation comparing study arm 1 and study arm 3 was not performed because it was expected that the difference between the two groups would always be larger than any other comparison. In all sample size calculations, level of significance was assumed at 5% and power was at a minimum of 80%. Groups 1 and 2 sample sizes were calculated based on equivalence test. It was assumed that 5% was the limit of equivalence, i.e. any difference bigger than 5% made the two groups not equivalent. If there were no difference between study arm 1 and study arm 2, 5% limit of equivalence, n=475 per group for 80% power to detect difference of more than 5%. 475 per group ANC clients were required to be 80% sure that the limits of a two-sided 95% confidence interval were excluded in difference between the two groups of more than 5%. Study arm two and study arm three sample sizes were calculated by assuming that arm 3 would have an uptake of partner HIV testing of 20%, while study arm 2 would reach at least 11% (the upper limit of equivalence). Based on these, 250 ANC clients were required to have an 80% chance of detecting, as significant at the 5% level, an increase in the partner HIV testing measure from 11% in study arm 2 to 20% in study arm 3. To avoid imbalance between the study groups, study recruited a similar number of participants for study arm 3, as in Groups 1 and 2. Since a higher sample size is required for the equivalence test to achieve greater statistical power to evaluate a possibly superior HIV testing rate in study arm 3 compared to Groups 1 or 2. In addition, the increased sample size allowed comparable statistical power in each group to evaluate heterogeneity of effects (i.e. effect modification). SAS software system was be used to calculate the sample size using the power procedure Pearson chi-square test for two proportions. At the facility levels, each facility was allocated sample size based on first ANC visits as per the year 2014 (KDHS data). Women were to be recruited until each randomized group had 475 women. For each ANC mother meeting eligibility criteria and consenting to participate, the intervention itself (for two of three study groups) were added to the end of the standard first ANC visit.

**Data collection:** quantitative data was collected using pre-tested questionnaires, which were administered at baseline and endline (12 weeks) to generate data on both primary and secondary outcomes (i.e. GBV, invalid tests, linkages to care) of the study. Clients were informed that the study was about HIV testing among male partners and that information and support about male partner testing may be given to each participant. Only women in study arm 3 were informed about the self-test kits. This was done in a private space at the clinic, immediately after determining eligibility.

**Male partner follow up:** the study sought out all male partners of ANC clients for an in-person survey by visiting the man at a place of their convenience e.g. at home or where they preferred, and this occurred 3 months from the date that each ANC client enrolled. During the recruitment of the ANC client, the research assistant explained that we would also like to talk to the male partner regarding HIV testing in general. If the woman was willing to let the research assistant contact her partner then her partner's telephone number or direction to their residence were captured. After the consent, the research assistant proceeded with the interviews. Male partners consenting to be interviewed were administered a structured questionnaire collecting information on socio-demographic characteristics, HIV testing history and whether the male partners had tested in the last three months, among other operational challenges in using the test kits.

**Figure 1:**
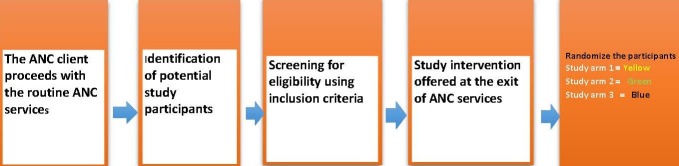
Study orocess

**Study process:** the ANC client proceeded with the routine ANC services as normal. Thereafter the research assistant identified potential participants and screened them for eligibility using a standardized screening tool. Eligible clients were invited to participate in the study and informed consent was sought. After recruitment the baseline questionnaire was administered and then participants were randomized into three study arms. Randomization of participants to the study arms was at a 1:1:1 ratio, using a computer-generated randomization list. Written allocation of assignments were sealed in individual opaque envelopes marked in different colours (yellow, green and blue for arms 1, 2 and 3). The envelopes for all study groups were put in a box and mixed well. During randomization, the consenting ANC client was requested to pick one envelope from the box and open to determine the assigned group. Figure 1 illustrates the study process.

## Results

**Number of women, men and couples recruited and followed up in each of the three study arm:** between May and October 2016, 2320 pregnant women were screened at the ANC clinic of which 1640 (71%) were eligible for the study and 1410 (87%) recruited and randomized into three study arms. Out of the 1410 recruited and randomized; 1217 (86%) of the women were successfully followed up during end line (three months later). The study also followed up 1,033 men and 1,107 couples after three months. Each of the three arms had a target sample size of 475 women at enrolment (totaling 1425 for all the study arms). However, in arm one, 471 (99.2 %) women were enrolled, of these women, 408 (86.6 %) were followed up with 375 (79.6%) of men and 366 (77.7%) of couples reached in arm one. In arm two, 467 (98.3%) of women were recruited of which 387 (82.9%) were followed, 77.5% of men also followed up and 352 (75.4%) of couples reached. In arm three, 472 (99.4%) of the women were recruited of which 422 (89.4%) were followed up and 396 (84.0%) and 389 (82.4%) of men followed up and couples reached, respectively. Table 1 presents the participants (women, men and couples) recruited and followed up in each of the three study arms.

**Table 1:** Number of women, men and couples recruited and followed up in each of the three study arms

Target Sample size	Control 1 N = 475 n (%)	Control 2 N = 475 n (%)	Intervention N = 475 n (%)	Total 1425 n (%)
No of Women recruited	471 (99%)	467 (98%)	472 (99%)	1410 (99%)
Number of women followed up	408 (87%)	387 (83%)	422 (89%)	1217 (86%)
Number of men recruited	375 (80%)	362 (78%)	396 (84%)	1033(80%)
Complete pairs of man and woman followed up	366 (78%)	352 (75%)	389 (82%)	1107 (79%)

**Baseline demographic data:** Table 2 and Table 3 presents participants characteristics across the three study arms (women and men). These characteristics were well balanced across the three study arms except for the testing history and the educational level by the male partner (P=0.009 and P=0.006 respectively).

**Table 2:** Demographic characteristics of women enrolled in the study by study arms (N=1410)

Demographic Characteristics	Arm 1 n (%)	Arm 2 n (%)	Arm 3 n (%)
**Level of Education**			
Primary	279 (59.2)	265 (56.7)	247 (52.3)
Secondary	192 (40.8)	202 (43.3)	225 (47.7)
**Religion**			
Catholic	83 (17.6)	103 (22.1)	107 (22.7)
Protestant	382 (81.1)	355 (76.0)	359 (76.1)
Other	6 (1.3)	9 (1.9)	6 (1.3)
**Employment**			
Self-employed	160 (34.0)	159 (34.0)	145 (30.7)
Employed	70 (14.9)	74 (15.8)	83 (17.6)
Unemployed	241 (51.2)	234 (50.1)	244 (51.7)
**Marital status**			
Single	5 (1.1)	9 (1.9)	8 (1.7)
Cohabitating	58 (12.3)	49 (10.5)	54 (11.4)
Currently married	408 (86.6)	409 (87.6)	410 (86.9)
**Age (years)**			
18-34	430 (91.3)	430 (92.1)	419 (88.8)
≥ 35	41 (8.7)	37 (7.9)	53 (11.2)

**Table 3:** Demographic characteristics of men who were followed up by the study arms

Characteristics	Arm 1 n (%)	Arm 2 n (%)	Arm 3 n (%)	X^2^Test P value
**Education**				
Primary	168 (44.8%)	151 (41.7%)	134 (33.8%)	0.006**
Secondary	207 (55.2)	211 (58.3)	262 (66.2)	
**Religion**				
Catholic	120 (32.0)	106 (29.3)	109 (27.5)	0.614
Protestant	241 (64.3)	246 (68.0)	272 (68.7)	
Other	14 (3.7)	10 (2.8)	15 (3.8)	
**Employment**				
Self-employed	183 (48.8%)	182 (50.3%)	188 (47.5%)	0.07939
Employed	160 (42.7)	147 (40.6)	189 (47.7)	
Unemployed	32 (8.5)	33 (9.1)	19 (4.8)	
**Marital status**				
Cohabitating	41 (10.9%)	43 (11.9%)	42 (10.6%)	0.8424
Currently married	334 (88.8)	319 (87.8)	354 (89.4)	
**Age**				
18-34	273 (72.8%)	268 (74.0%)	272 (68.7%)	0.4042
35 and above	96 (25.6%)	92 (25.4%)	114 (28.8%)	

**HIV testing acceptability rates among male partners:** in this study, 83% (327) of the males in arm three where test kits were provided to the ANC mother accepted testing for HIV as compared to those in arms two and one 37% (133) and 28% (106) respectively.

**Males testing history across the three study arms:** prior to the study, slightly more men in arm three had ever tested for HIV before (92% compared to 86% in arm two and 84% in arm one) and this was statistically significant (p-value=0.009). A three-month follow-up questionnaire was administered to assess whether men reported testing for HIV during the intervention. In the self-testing arm three, 82.6% tested for HIV in the three months after the woman's ANC visit compared to only 37.0% in the enhanced control group (arm 2) and 28.3% in the standard control group (arm 1). This was statistically significant (p-value <0.001). In relation to location of HIV testing, 85% of men in the intervention arm took the test at home, while nearly everyone in the control arms went to the Clinic/VCT center. All the respondents in arm 3 who tested at home used the HIV oral self-test kits provided to the ANC clients. One male partner in arm 2 bought the test kit to self-test. Seventy-two per cent (204) of men who took an HIV test at home did not take a confirmatory test.

**Male partner testing against ANC mother characteristics across the study arms:** in arm one (control), marital status (P<0.001), and wealth status (P<0.001) of the woman played a significant role in determining male partner testing. In arm two, however it was the level of education and wealth status of the woman that determined testing by the male partner (P=0.027 and 0.005) respectively. In arm three it was the age of women that significantly determined testing by the male partner (P= 0.04), 81% of male spouses of women ages 18-34 years tested as compared to 19% in the same age bracket. In addition, more male spouses (67%) of women who were 35 years and over tested for HIV.

**Determinants of HIV testing among male partners (as reported by men):** to establish determinant of HIV testing among male partners, we assessed age, study arm, employment, religion, wealth index and education. At bivariate analysis, study arm, education and wealth index were associated with HIV testing. Men in arm 3 were 12 times more likely to test for HIV compared to men in arm 1 (OR 11.86; 95% CI 7.00, 20.10). Men with secondary education and above were two times more likely to test for HIV compared to men with primary education (OR 2.21; 95% CI 1.76, 2.76). As the wealth index increased from second lowest, second highest and to highest, the likelihood of testing for HIV reduced by 30%, 51% to 58% respectively compared to men in the lowest wealth index. After adjusting for other factors (age, study arms, employment, religion, wealth index and education), only study arm and education were associated with testing for HIV. Men in study arm 3 were almost 13 times likely to test for HIV compared to men in arm 1 (OR 12.45; 95% CI 7.35,21.08) and men with secondary education and above were two times more likely to test for HIV compared to men with primary education (OR 2.05; 95% CI 1.74, 2.42). Table 4 presents the adjusted odds ratio results.

**Table 4:** Determinants of HIV testing among male partners

	Un-Adjusted		Adjusted		P Value
	Odds ratio	95% CI	Odds ratio	95% CI	
**Age**					
18-24	1.00		1.00		
25-34	1.16	0.72 1.88	0.99	0.57 1.69	P > 0.005
≥35	1.31	0.63 2.73		0.55 2.42	
**Study arm**					
Arm 1	1.00		1.00		
Arm 2	1.58	1.19 0.57	1.58	1.25 2.00	P < 0.005
Arm 3	11.86	7.00 20.10	12.45	7.35 21.08	
**Employment**					
Employed	1.00		1.00		
Un-Employed	0.63	0.40 1.01	1.09	0.56 2.02	P > 0.005
Self-Employed	0.87	0.64 1.16	1.09	0.88 1.35	
**Religion**					
Catholic	1.00		1.00		
Other	1.19	0.72 1.98	1.21	0.68 2.15	P > 0.005
Protestant	1.17	1.02 1.35	1.04	0.88 1.23	
**Wealth Index**					
Lowest	1.00		1.00		
Second Lowest	0.70	0.49 1.00	0.84	0.53 1.31	
Second Highest	0.49	0.32 0.75	0.64	0.37 1.13	P > 0.005
Highest	0.42	0.24 0.76	0.57	0.28 1.14	
**Education**					
Primary	1.00		1.00		
Secondary (A or O level)	2.21	1.76 2.76	2.05	1.74 2.42	P < 0.005

### Effectiveness of HIV oral self-testing kit and improved invitation letter in increasing couple testing rates

**Women who reported discussing HIV and tested together with their partners:** almost all women discussed HIV testing with partner, with those in arm three being 98% (413), arm two 96% (371) and arm one 97% (395). In arm three, 79% (334) of women reported to have taken an HIV test together with the partner. About 35% (136) and 27% (110) of women reported testing together in arms two and one, respectively. ANC clients and their partners were separately asked if they had tested together or knew whether their partner had been tested. There was a strong agreement in all the three arms according to the Kappa agreement statistical analysis (Cohen's Kappa statistic, 0.90 (0.86-0.95), P-value<0.001).

Prevalence of gender based violence (GBV), intimate partner violence (IPV), gender inequity and other harm associated with introduction of oral HIV self-testing on male partner testing: on further investigation to establish prevalence of GBV, IPV, gender inequity and other social harm associated with the introduction of self-testing on males among women in the study, there was no incidence of GBV or IPV reported during the study period. Further there were no significant differences across the arms in terms of men’s reactions towards GBV and IPV. Generally, over 80% of men interviewed in the three study arms did not support any form of gender-based violence.

### HIV testing among ANC clients

**Person conducting HIV test at home:** all women in arm one who reported testing, tested in the clinics or VCT centers. Among those women in arm two and three who tested for HIV at home, 100% [5] and 99.7% (310) respectively self-tested and they did not involve a counsellor to conduct the test.

**Effectiveness of location of HIV testing in improving couple testing:** a total of 560 couples tested together for HIV with 321, 133, and 106 couples testing together in arm 3, 2, and 1 respectively. In arm one and two 99.1% (105) and 97.7% (130) of the couples tested at clinic/VCT respectively. In arm 3, 86.6% (278) of the couples tested at home. Home testing was statistically significant in improving couple HIV testing (p-value<0.001).

**Figure 2:**
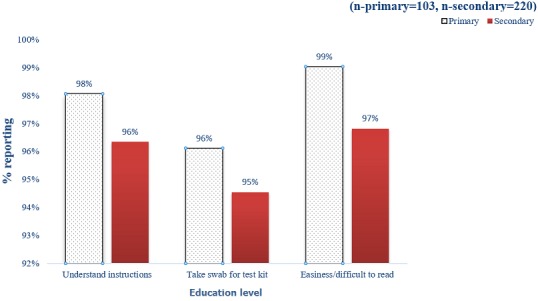
Education level and use of test kits (arm three)

**Figure 3:**
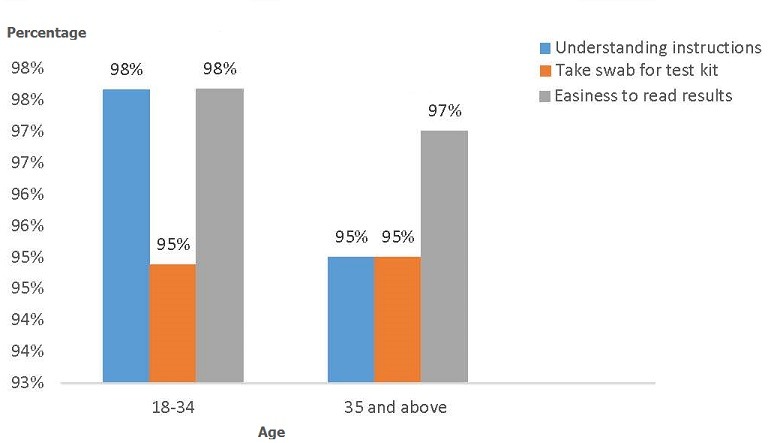
Age and the use of test kits

**Usability of test kits by the ANC mothers and their partners:** usability of test kits by ANC mother in arm three together with their partners was assessed using three parameters; understanding of user instructions for HIV self-testing kits, reading test results and taking the cheek swab. Participants with primary and secondary school education and those aged 18-34 years and 35 years and older reported it was very easy to understand user instructions for HIV self-testing kit, read test results and take the cheek swab as illustrated in Figure 2 and Figure 3.

## Discussion

The study finding showed that there were significant differences in testing rates (P=0.01) between arm one and two while arm three was superior to arm two with significantly higher testing rates (P<0.001). There were high testing and acceptability rate among the men who got the test kit and the improved partner invitation letter in arm three compared to other two study arms (83%, 37% and 28% respectively). KAIS 2012 showed overall, 71% of respondents would be willing to use such a kit if it were to be available to them, more men (74%) than women (67%) indicated willingness to use an HIV self-test kit [1]. This study findings aligns with existing literature on HIV self-testing, which suggest users (including the general population) may prefer oral fluid-based HIV Rapid Diagnostic Test (RDT) to finger stick/whole blood-based HIV RDT because they are reportedly easier to perform and are perceived as less painful as long as they are provided with sufficient HIV prevention information [17]. This will provide an opportunity for HIV testing among clients who dislike needle pricks. Male partners of clients in arm three (those provided with improved letter and self-testing kits) were twelve times more likely to test for HIV compared to men in arm one (control). During enrolment women in arm three and two got brief counselling sessions on how to counsel the partner, discuss HIV and demonstrate the kits use to the partner. Several studies have demonstrated that when given the opportunity to participate in sexual and reproductive health programmes, such as family planning and the PMTCT programmes, men preferred to be positively involved in promoting the health of their families and communities [9]. There were no incidence of GBV or IPV reported during the study period. In arm three where a test kit was provided, over 80% of the male partners did not support any form of GBV or IPV. There were several operational challenges experienced by the ANC mothers during the study period; in arm three only 79% of the couples actually tested together. Going for confirmatory testing after the initial HIV screening test (using the oral HIV test kit) is very important to confirm a true positive or a true negative test result, however in this study only 28% of the participants who took a HIV test at home went for a confirmatory test at the health facility. This indicates the need for emphasis on importance of going for confirmatory testing after initial HIV screening. Age and educational level of the study participants did not affect usability of the test kits (reading instructions, taking the cheek swab and interpreting the results).

## Conclusion

Giving ANC mothers test kits and improved male invitation letter to test together with their male spouses increased the odds of male partner testing by twelve times compared to the control arm. Couple testing was also increased by 80% in the five counties compared to the national figure which stands at 6%. Despite the improved couple testing rates using the self-testing kits at home, there still remains a big challenge of referring the male partner to the facility for confirmatory testing.

### What is known about this topic

- Women have greater access to HTC services than their male partners due to their more frequent contact with health services;
- Correlates and other characteristics of male HIV testing behavior and the reasons for obtaining HIV tests have been demonstrated and explained in sub-Saharan Africa using population-based studies and within workplace and clinic-based HTC trial;
- Engagement and enrolment of male partners remains a challenge in scaling up couples services. Men are less likely than women to test for HIV.

### What this study adds

- The finding from this study could inform how best to close the gap in HIV testing using self-testing kits and possibly inform on how to achieve the first 90 of the WHO's 90-90-90 global HIV targets. Specifically the study provides new ways of addressing poor testing rates among male partners of ANC clients. The findings could further inform HIV programs and policy makers on the best way to improve low testing rates among partners of ANC clients and also demonstrates how self-testing may provide males who are not currently reached by HTS an opportunity to test in private.

## Competing interests

The authors declare no competing interests.
